# Supplementary material for: Outlier detection in state-space models using mean-shift penalisation
Source: Stat Comput. 2026 Jul 13;36(4):176. doi: 10.1007/s11222-026-10935-4 (PMC13364811; doi:10.1007/s11222-026-10935-4)
Supplement: Supplementary file 1 — (pdf 468 KB) [file 11222_2026_10935_MOESM1_ESM.pdf]

# Appendix

**Table A1**: Comparison of standard Kalman recursions and the missing-observation Kalman recursions. When a timepoint is detected as an outlier, then $\gamma_t \neq \mathbf{0}$, resulting in the observation being treated as missing and $\boldsymbol{K}_t = \mathbf{0}$.

| Term | Standard recursion | Missing-observation recursion |
|---|---|---|
| $\boldsymbol{x}_{t\|t-1}$ | $\boldsymbol{\Phi}\boldsymbol{x}_{t-1\|t-1}$ | $\boldsymbol{\Phi}\boldsymbol{x}_{t-1\|t-1}$ |
| $\boldsymbol{P}_{t\|t-1}$ | $\boldsymbol{\Phi}\boldsymbol{P}_{t-1\|t-1}\boldsymbol{\Phi}^\top + \boldsymbol{\Sigma}_{\boldsymbol{w}}$ | $\boldsymbol{\Phi}\boldsymbol{P}_{t-1\|t-1}\boldsymbol{\Phi}^\top + \boldsymbol{\Sigma}_{\boldsymbol{w}}$ |
| $\boldsymbol{y}_{t\|t-1}$ | $\boldsymbol{A}\boldsymbol{x}_{t\|t-1}$ | $\boldsymbol{A}\boldsymbol{x}_{t\|t-1}$ |
| $\boldsymbol{S}_{t\|t-1}$ | $\boldsymbol{A}\boldsymbol{P}_{t\|t-1}\boldsymbol{A}^\top + \boldsymbol{\Sigma}_{\boldsymbol{v}}$ | $\boldsymbol{A}\boldsymbol{P}_{t\|t-1}\boldsymbol{A}^\top + \boldsymbol{\Sigma}_{\boldsymbol{v}}$ |
| $\boldsymbol{K}_t$ | $\boldsymbol{P}_{t\|t-1}\boldsymbol{A}^\top\boldsymbol{S}_{t\|t-1}^{-1}$ | $\boldsymbol{P}_{t\|t-1}\boldsymbol{A}^\top\boldsymbol{S}_{t\|t-1}^{-1} \times 1_{\{\boldsymbol{\gamma}_t = \mathbf{0}\}}$ |
| $\boldsymbol{x}_{t\|t}$ | $\boldsymbol{x}_{t\|t-1} + \boldsymbol{K}_t(\boldsymbol{y}_t - \boldsymbol{y}_{t\|t-1})$ | $\boldsymbol{x}_{t\|t-1} + \boldsymbol{K}_t(\boldsymbol{y}_t - \boldsymbol{y}_{t\|t-1})$ |
| $\boldsymbol{P}_{t\|t}$ | $\boldsymbol{P}_{t\|t-1} - \boldsymbol{K}_t\boldsymbol{A}\boldsymbol{P}_{t\|t-1}$ | $\boldsymbol{P}_{t\|t-1} - \boldsymbol{K}_t\boldsymbol{A}\boldsymbol{P}_{t\|t-1}$ |

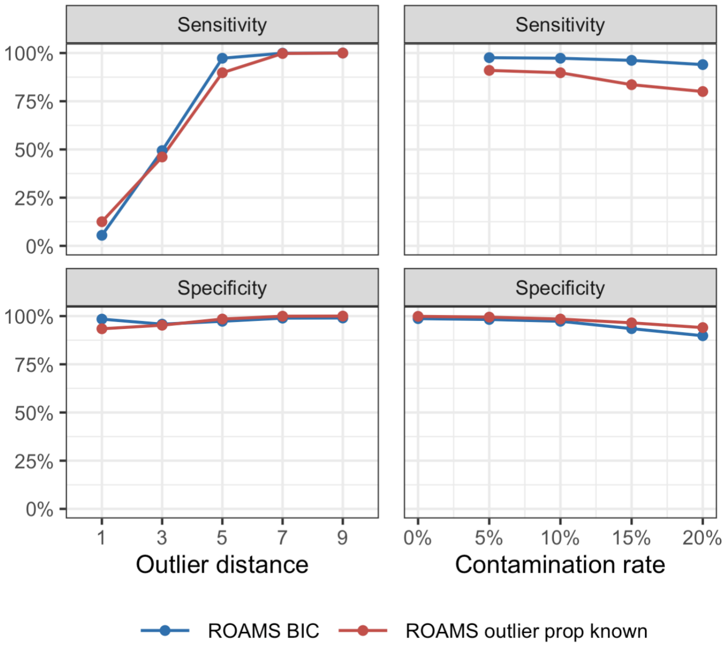


**Fig. A1**: Sensitivity and specificity, averaged across the simulation runs, for 10% contamination across different outlier distances (left) and outlier distance 5 across different contamination rates (right), for BIC-selected $\lambda$ and for true-contamination-rate-informed $\lambda$.
